# In Simplici Salus

**Published:** 1891-08

**Authors:** 


### "In Simplici Salus."

Hippocrates proclaimed that "accurate observation of facts, and correct generalization from them, forms the only rational basis of medicine," and so we are taught to-day. To discover truth in science, the most learned will admit, is very often difficult, but in no science is it more difficult than in medicine. Independent of the common defects of medical evidence, our self-interest, our self-esteem, our prejudices, our likes and dislikes, and not infrequently our ignorance, only too often hide the truth from our view, and we ascribe too much to art, and too little to the operations of nature. Thus the mass of testimony is most with art, and although we believe we are right in our reasoning, we only pursue the old, time-honored course that has been instilled into our minds through training and education. The best and safest practitioner is he who knows when to abstain from acting as well as when to act; in other words, who has learned when and to what extent the case can be left to the salutary processes of nature. The tendency to recovery which manifests itself under different modes of treatment, and even in spite of opposite modes, has induced in some minds a degree of skepticism as to the utility of any remedies. That the opposite error to that of mischievous or meddlesome activity may likewise be easily carried far is at once apparent. It does not follow because the majority of diseases, such as continued fevers and acute affections generally get well, with or without the administration of medicine, that therefore the disease should be abandoned to what Cullen calls the "*vis medicatrix naturae.*"

A knowledge of the circumstances upon which health depends is one of the most important parts of the moral and intellectual education of the true physician. The essentials necessary to the attainment of health are:

1. The inheritance of a healthy constitution.
2. Pure atmosphere and water.
3. Wholesome food in quantity and quality.
4. Freedom from contagious and infectious diseases.

Either of these primitive essentials of health is controlled but little, if any, by the individual efforts of man. To medical science solely must mankind look for the foundation rock from whence the principles governing these essentials of nature's law are to be revealed by virtue of intelligent, progressive, active, zealous and truly conscientious physicians, who will, sooner or later, succeed in educating the legislator to understand and realize the fact paramount that only through the State or municipal government can we ever hope to see mankind enjoy these blessings. Nature puts into our hands the means of preserving health and this gift involves responsibility. Health will be counted among those talents for the use of which we are to answer to our Creator, and it is our duty to become fully acquainted with the laws which regulate and govern it.

Experience teaches that disease, as well as health, is controlled by nature, that her laws must be consulted if we would practice successfully. But alas! how often do we find—even in this most enlightened age of science—the truth as expressed by the late Prof. Chapman, "That many physicians are given to profound thought, and possess extensive knowledge, united with sterling honesty, being by nature endowed with the highest order of talents, and yet be wanting in good common sense." The most experienced, close observing, earnest searchers after truth in nature's operations, most skillful and best physicians, hesitate above all things to give large quantities of medicine, and proclaim the best way to help the invalid to health is simply to "assist nature." The writings of Drs. Benjamin Rush, Shippen, Chapman, Radcliffe, Bostwich, Dumoulin, and Oliver Wendell Holmes, Sir Astley Cooper, Sir Wm. Gull, and numerous other shining lights, are too well known to need reiteration, but are simply called to mind as evidence that the more matured minds in the profession are guided by the light of nature's *in simplici salus*.—Editorial in *Amer. Asso. Jour. of Medicine*.

